# High glucose-induced injury in human umbilical vein endothelial cells is alleviated by vitamin D supplementation through downregulation of TIPE1

**DOI:** 10.1186/s13098-024-01264-5

**Published:** 2024-01-13

**Authors:** Zhoujun Liu, Haogang Sun, Yu Chen, Jia He, Lin Zhu, Bing Yang, Wenzhuo Zhao

**Affiliations:** 1 Department of Endocrinology, Suzhou Wuzhong People’s Hospital, 61 Dongwu North Road, Suzhou, Jiangsu 215000 China; 2 Department of Obstetrics, The First Affiliated Hospital of Shihezi University, Shihezi, Xinjiang China; 3 Endocrine Research Center, Affiliated Hospital of Integrated Traditional Chinese and Western Medicine, Nanjing University of Chinese Medicine, Nanjing, China; 4 Department of Public Health and Key Laboratory of Xinjiang Endemic and Ethnic Diseases of the Ministry of Education, School of Medicine, Shihezi University, Shihezi, Xinjiang China; 5 Department of Pediatrics, The First Affiliated Hospital of Shihezi University, Shihezi, Xinjiang China

**Keywords:** Vitamin D, High glucose, Human umbilical vein endothelial cells, TIPE1

## Abstract

**Background:**

Diabetes mellitus (DM) and its associated vascular complications have become a worldwide health concern. The effects and mechanism of vitamin D supplementation on endothelial function under high glucose condition remain elusive.

**Methods:**

Human umbilical vein endothelial cells (HUVECs) were treated with 35 mM glucose, then 100 nM vitamin D were added. Transwell migration assay, CCK-8, immunofluorescence, flow cytometry, autophagy flux and transmission electric microscope were performed.

**Results:**

Vitamin D reduced apoptosis, promoted migration and enhanced viability of HUVECs, decreased TIPE1 (Tumor necrosis factor-α-induced protein 8-like 1) under high glucose conditions. Overexpression of TIPE1 reverses the effects of vitamin D by increasing ROS production, inflammation, cell apoptosis, and suppressing autophagy, cell migration and viability. And vitamin D negatively correlated with TIPE1 mRNA level in DM patients.

**Conclusions:**

Vitamin D reverses the harmful effects of high glucose on HUVECs by reducing TIPE1 expression. And vitamin D supplementation could help to alleviate high glucose-induced injury in type 2 diabetes mellitus patients with microvascular complications.

## Introduction

As a global public health concern, diabetes mellitus (DM) and its associated complications pose a substantial threat to human well-being [1, 2]. Type 2 DM (T2DM) accounts for more than 90% of DM cases and is characterized by hyperglycemia, insulin resistance, and dysregulated lipid metabolism [3]. Patients with T2DM frequently experience microvascular complications, which arise from chronic exposure to hyperglycemia and result in detrimental effects on the microvasculature [4]. Notably, diabetic nephropathy, retinopathy, and neuropathy are common consequences of this damage, significantly impacting both quality of life and life expectancy [5, 6]. The role of endothelial damage in the development of microvascular complications of T2DM is of paramount importance [1].

Vitamin D is an essential nutrient for the human body, and its insufficiency has been found to be associated with T2DM [7]. Recent evidence has indicated that serum 1,25-OH-vitamin D (the biologically active form) is positively correlated with insulin sensitivity and secretion, while a deficiency in vitamin D is negatively linked to glycemic control [7, 8]. Furthermore, recent studies have suggested that individuals with a serum 1,25-OH-vitamin D level below 50 nmol/L have a higher incidence of macrovascular and microvascular events compared to those with a level above 50 nmol/L [9]. The role of vitamin D in the development of microvascular complications in T2DM remains uncertain. Consequently, this study sought to examine the impact and underlying mechanism of vitamin D supplementation on endothelial functions using a model of human umbilical vein endothelial cells (HUVECs) exposed to high glucose conditions.

Specifically, the study focused on the involvement of TIPE1 (Tumor necrosis factor-α-induced protein 8-like 1, TNFAIP8L1), a recently discovered member of the TIPE protein family known to contribute to inflammation, endothelial dysfunction, and atherosclerosis [10]. In this study, it was observed that the downregulation of TIPE1 played a role in mediating the effects of vitamin D in HUVECs (Human umbilical vein endothelial cells) under high glucose conditions.

## Methods

### Patients and ethics approval

The Ethics Committee of Suzhou Wuzhong People’s Hospital (KJ-2023-022-01) granted approval for this study. Prior to their participation, all patients were provided with information regarding the objective, content, and significance of the study. Informed consent forms were signed by all patients.

### Measurement of serum vitamin D level

A total of 40 type 2 patients with microvascular complications and 40 type 2 patients without complications were randomly selected for recruitment. Serum vitamin D levels were assessed using chemiluminescence enzyme immunoassay (IDS, Boldon, UK).

### Cell culture and treatment

Human umbilical vein endothelial cells (HUVECs) were purchased from American Type Culture Collection (ATCC, CRL-1730, USA) and cultured in endothelial cell growth medium (ScienCell, USA). Experiments were conducted on cultured HUVECs in their 3rd-6th passage. HUVECs were treated with 35 mM glucose (G8769, Sigma, USA) for 12 h to simulate cells exposed to hyperglycemia. 100 nM vitamin D (1α,25-dihydroxyvitamin D3, D1530, Sigma, USA, dissolved in ethanol) was added in the culture medium of HUVECs for 24 h after pretreatment with 35 mM glucose for 12 h. HUVECs incubated with a normal concentration of glucose (5.6 mM) served as control.

### Cell viability test

Cell counting kit 8 (CCK-8) kit (Vazyme, Nanjing, China) was used to detect HUVECs cell viability with different treatments. The absorbance was measured at 450 nm on a microplate reader after 1.5 h of incubation of 10 µL CCK-8 solution at 37 °C (Molecular Devices, CA, USA).

### Terminal deoxynucleotidyl transferase dUTP nick end labeling staining

Terminal deoxynucleotidyl transferase dUTP nick end labeling (TUNEL) assay kit (Roche Molecular Biochemicals, Mannheim, Germany) was used. The stained cells were imaged with a microscope (Observer D1, Zeiss).

### Cell migration transwell analysis

Cell migration transwell assay was performed to check the migration ability of HUVECs with different treatments. Treated HUVECs were seeded into the upper chamber in serum-free medium, culture medium containing FBS were added to the lower chamber. After 24 h, migrated cells were stained with 0.1% crystal violet reagent solution (Beyotime, China) for 15 min and imaged with a microscope (Observer D1, Zeiss).

### Reverse transcription-quantitative PCR (RT-qPCR)

The expression levels of TIPE1 mRNA were evaluated by RT-qPCR using beta-actin as the internal control following routine procedures. The following primer sequences were used for RT-qPCR: TIPE1 forward, 5’-GGACTTGGCCTCAGTTTTGC-3’, and reverse, 5’-GCCTTGGACGCCATCTTACT-3’; and beta-actin forward, 5’-TCACCATGGATGATGATATCGC-3’, and reverse, 5’-ATAGGAATCCTTCTGACCCATGC-3’.

### Western blot and immunofluorescence staining

The proteins were extracted from indicated treated HUVECs using RIPA lysis buffer containing protease and phosphorylase inhibitors. Then equivalent proteins were separated by SDS-PAGE (sodium dodecyl sulfate polyacrylamide gels) and then transferred to a PVDF (polyvinylidene difluoride) membrane (Millipore, USA). After blocking, the membranes were incubated with TIPE1 antibody (ab85409, Abcam, USA) and then horseradish-peroxidase conjugated secondary antibodies. The membrane was detected using ECL (enhanced chemiluminescence) solution. Beta-ACTIN was used as the internal control.

In immunofluorescence staining, cells were fixed, blocked with 30% goat serum, and incubated with TIPE1 antibody (CSB-PA837859LA01HU, CUSABIO, China). Following PBS washing, cells were incubated with secondary antibody Alexa Fluor 488-congugated AffiniPure Goat Anti-Rabbit IgG(H + L) and counterstained with DAPI. An Olympus optical microscope or confocal laser scanning microscopy was used to detect and analyze images.

### Analysis of cellular reactive oxygen species (ROS) levels

Intracellular reactive oxygen species (ROS) production was quantified by a ROS assay kit (Beyotime, Shanghai, China). Treated HUVECs were incubated with media containing 10 µM DCFH-DA at 37 °C for 15 min. DCFH-DA is converted into dichlorodihydrofluorescein (DCFH) by oxygen free radicals, showing green fluorescence, as imaged using fluorescence microscopy.

### Overexpression of TIPE1

The overexpression plasmids of TIPE1 was constructed by Sangon (Shanghai, China). The vector was used as the negative control. Plasmids were transfected using Lipofectamine 3000 (Thermofisher, USA) according to the manufacture’s protocol.

### Cell apoptosis assay

Apoptosis of HUVECs was quantified using the Annexin V-FITC/propidium iodide assay. Treated HUVECs were digested with 0.25% trypsin, stained with Annexin V-FITC and PI (Vazyme, Nanjing, China) following the manufacturer’s instructions and analysed by flow cytometry (Beckman Coulter, USA) and Kaluza software.

### Autophagy flux and transmission electron microscopy

HUVECs were transfected with mCherry-GFP-LC3B using Lipofectamine 3000 (Thermofisher, USA). After 4 h, cells were incubated with different treated medium overnight. Later, cells were fixed with 4% paraformaldehyde, mounted on slides and analyzed by confocal laser scanning microscope.

### Statistics

Three independent replicates were used to determine the mean and standard deviation. Data analysis and presentation were performed using IBM SPSS 22.0 software (New York, USA) and GraphPad Prism 8.0 (San Diego, CA, USA). Student’s *t* test was used to compare outcomes between two groups. For comparisons of three or more groups, one-way repeated measures (RM) analysis of variance (ANOVA) was performed. *P*-values of 0.05 constituted statistical significance. The association between TIPE1 mRNA and serum vitamin D level was performed using Pearson correlation analysis by GraphPad Prism 8.0.

## Results

### Serum vitamin D level was lower in diabetes mellitus patients with microvascular complications

**Fig. 1:**
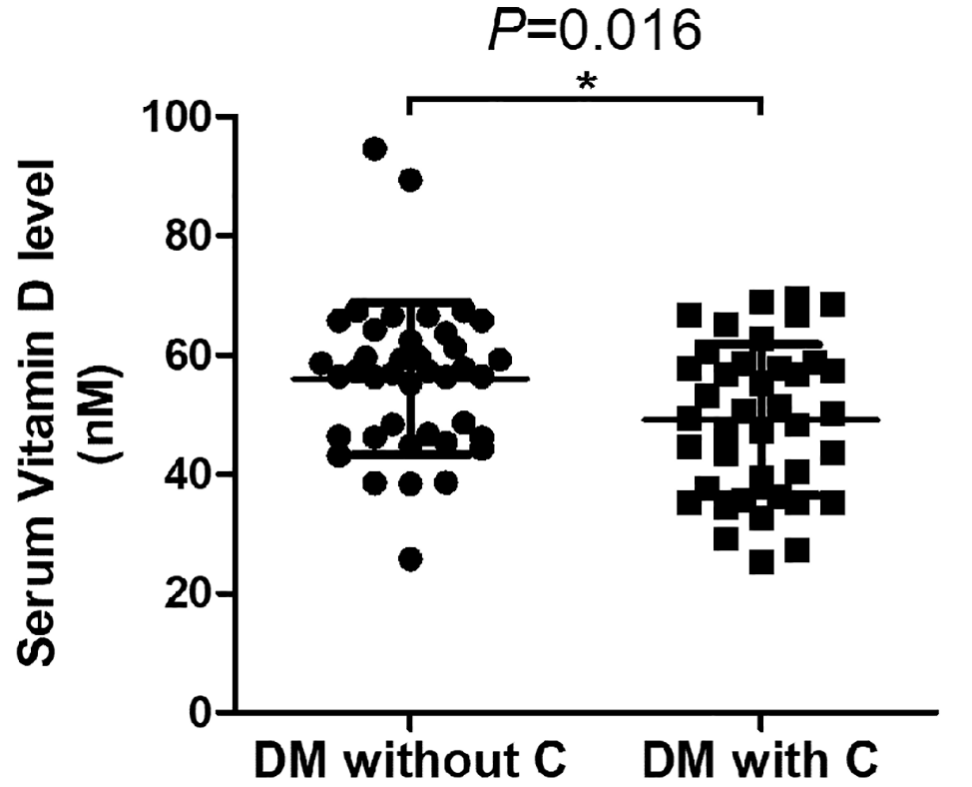
Vitamin D level is significantly lower in type 2 diabetes mellitus patients with microvascular complications. DM without C, diabetes mellitus patients without complications; DM with C, diabetes mellitus patients with microvascular complications. *P<0.05

The serum of 40 patients diagnosed with type 2 diabetes without complications and 40 patients diagnosed with type 2 diabetes with microvascular complications, including diabetic cardiomyopathy, nephropathy, retinopathy, and peripheral neuropathy, was collected in a random manner. The clinical information of the patients was recorded in Table 1. The measurement of vitamin D level was conducted using routine chemiluminescence enzyme immunoassay. The findings indicated a significant decrease in serum vitamin D level among diabetes mellitus patients with microvascular complications in comparison to those without complications, as illustrated in Fig. 1. Based on this phenomenon, it is postulated that a diminished level of serum vitamin D may be linked to the occurrence of microvascular complications in individuals with diabetes mellitus. Consequently, our objective is to investigate the impact and mechanism of vitamin D under conditions of elevated glucose by employing HUVEC cell lines.

**Table 1:** Clinical information of patients whose serum vitamin D level were detected

Clinicopathologic features	DM without C	DM with C	*P* value
Gender (female)	40 (22)	40 (26)	0.49
Age (years)	54.37 ± 2.82	54.85 ± 2.46	0.42
BMI (body mass index)	31.88 ± 1.86	32.85 ± 1.90	0.02
Waist circumference (cm)	117.31 ± 7.77	122.51 ± 7.56	0.003
Serum vitamin D level (nM)	56.05 ± 12.80	49.09 ± 12.60	0.016
Diabetic cardiomyopathy (cases)		9	
Diabetic nephropathy (cases)		8	
Diabetic retinopathy (cases)		8	
Diabetic peripheral neuropathy (cases)		15	

DM without C, diabetes mellitus patients without complications; DM with C, diabetes mellitus patients with microvascular complications

### Vitamin D reduced cell apoptosis and increased the migration and viability of endothelial cells treated with high glucose

**Fig. 2:**
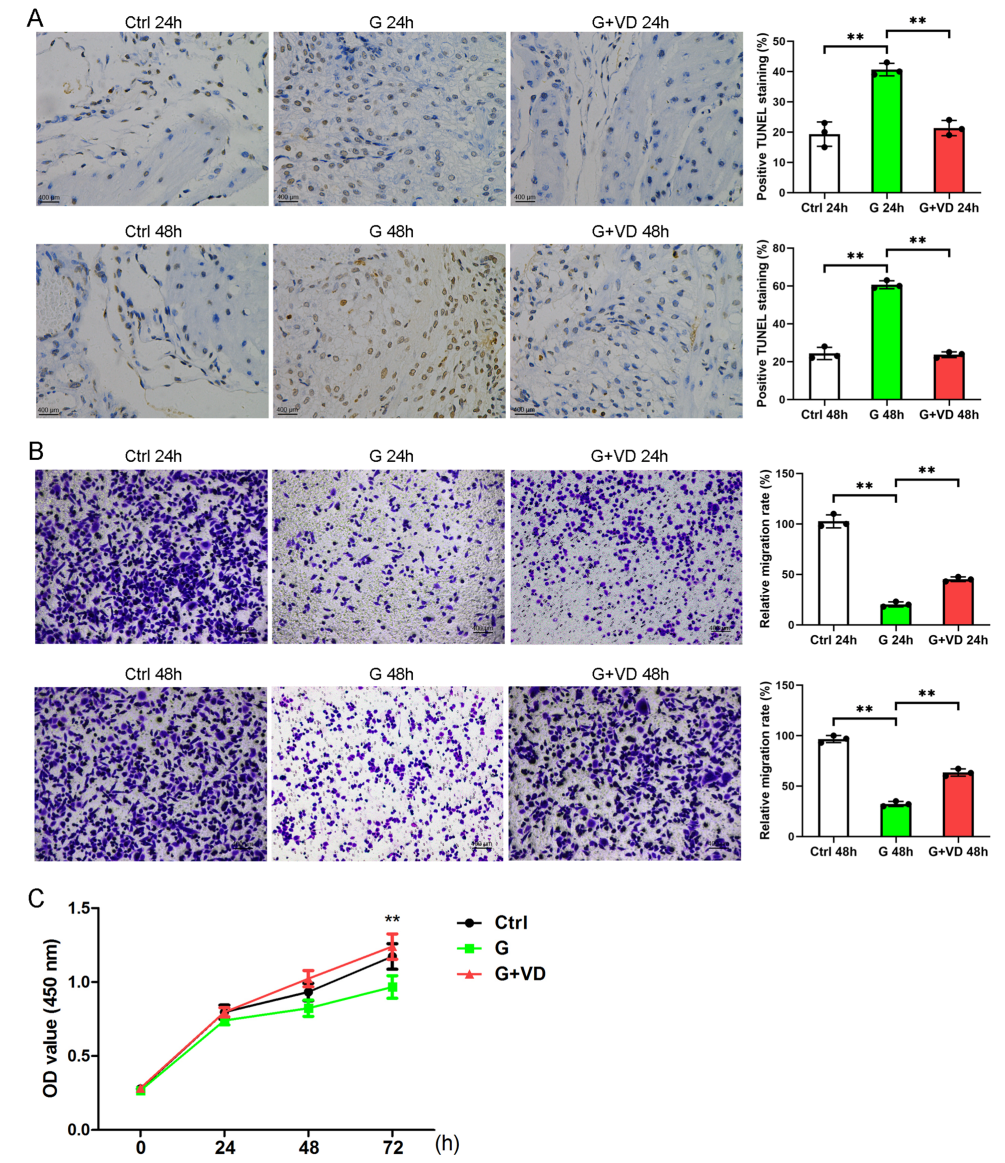
?Vitamin D inhibits cell apoptosis, promotes cell migration and viability in human umbilical vein endothelial cells treated by high glucose. (**A**) Cell apoptosis level was detected by TUNEL. (**B**) Cell migration was tested by Transwell assay. (**C**) Cell viability was analysed by CCK-8 assay. Ctrl, a normal concentration of glucose (5.6 mM); G, high glucose treatment; G+VD, high glucose plus vitamin D treatment. **P<0.01

Previous studies have demonstrated that the administration of glucose at a concentration of 35 mM induces notable cytotoxic effects [11–14]. Consequently, we subjected human umbilical vein endothelial cells (HUVECs) to a 12-hour treatment with 35 mM high glucose to simulate the exposure of endothelial cells to hyperglycemia. In the G + VD (Glucose + vitamin D) group, HUVECs were pretreated with 35 mM glucose for 12 h, followed by the addition of 100 nM vitamin D in the culture medium for 24 h. The used concentration of 100 nM vitamin D was previously reported to protect the high glucose induced injury [15, 16]. The TUNEL assay demonstrated that vitamin D reduced apoptosis levels in the presence of high glucose at both 24 and 48 h (Fig. 2A). Additionally, transwell migration analysis indicated that vitamin D promoted cell migration (Fig. 2B) and enhanced cell viability (Fig. 2C) in HUVECs exposed to high glucose.

### Vitamin D reduced the protein expression of TIPE1 under high glucose conditions in HUVECs

To investigate the downstream gene of vitamin D in the context of high glucose conditions, our focus was on TIPE1 (tumor necrosis factor α-induced protein 8-like 1, TNFAIP8L-1).

**Fig. 3:**
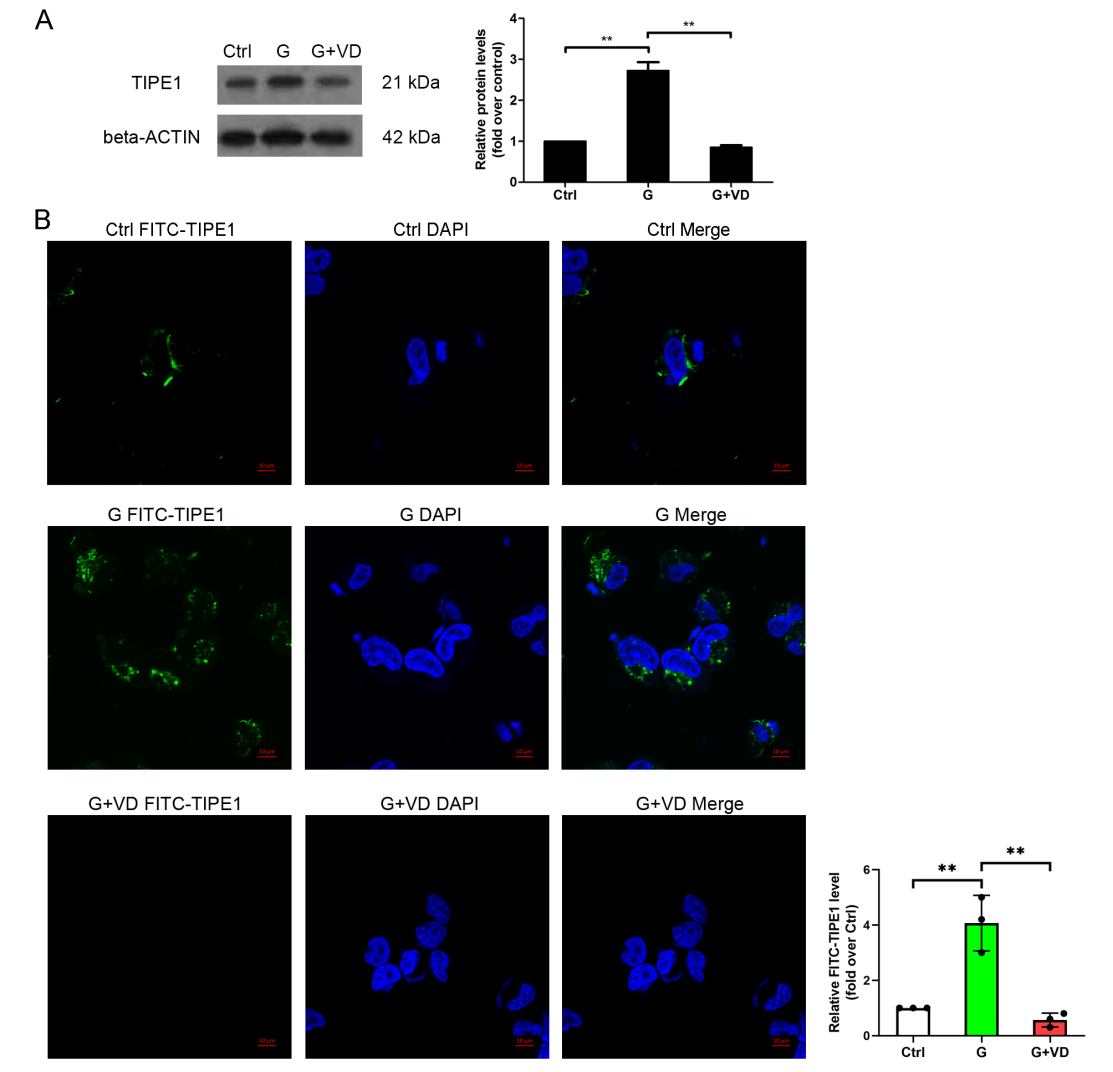
Vitamin D represses the expression of TIPE1 in human umbilical vein endothelial cells treated by high glucose. (**A**) TIPE1 protein expression was checked by Western blot. (**B**) TIPE1 protein expression was determined by immunofluorescence. Ctrl, a normal concentration of glucose (5.6 mM); G, high glucose treatment; G+VD, high glucose plus vitamin D treatment. **P<0.01

TIPE1, a member of the TNFAIP8 family, has been reported to play a crucial role in various cellular processes including cell death, inflammation, endothelial dysfunction, and atherosclerosis [10]. Notably, our experimental findings using western blot and immunofluorescence analysis demonstrated that vitamin D treatment resulted in a reduction in the protein expression of TIPE1 under high glucose conditions (Fig. 3A and B).

### Overexpression of TIPE1 reverses the effects of vitamin D under high glucose conditions by increasing ROS production, inflammation, cell apoptosis, and suppressing autophagy, cell migration and viability

**Fig. 4:**
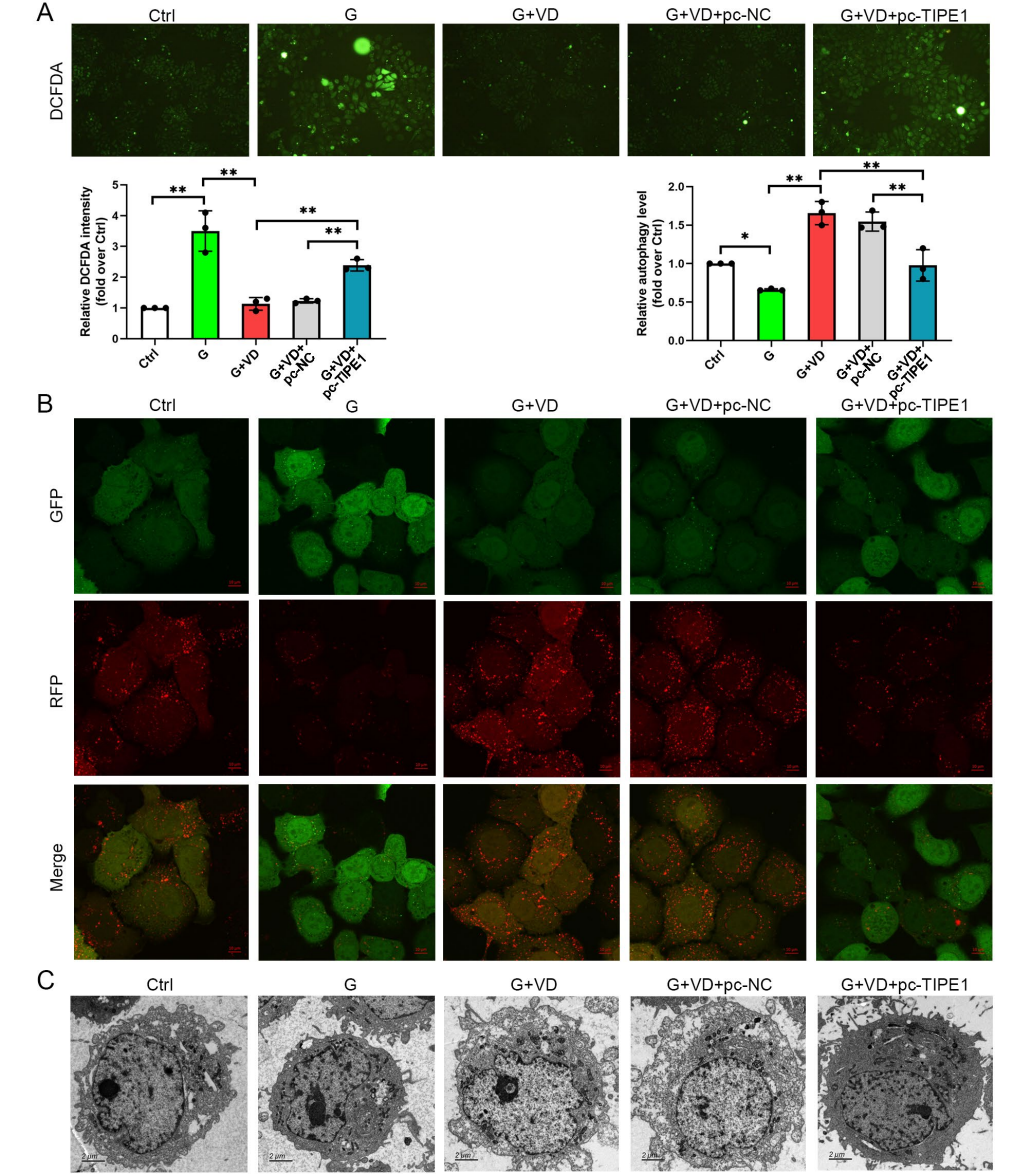
?Overexpression of TIPE1 reverses the effects of Vitamin D in ROS and autophagy in human umbilical vein endothelial cells treated by high glucose. (**A**) ROS level was detected by DCFH-DA. (**B**) Autophagy flux was tested by mCherry-GFP-LC3B assay. (**C**) Transmission microscope was performed to check the autophagosome. Ctrl, a normal concentration of glucose (5.6 mM); G, high glucose treatment; G+VD, high glucose plus vitamin D treatment; G+VD+pc-NC, high glucose plus vitamin D treatment as well as pc-NC transfection; G+VD+pc-TIPE1, high glucose plus vitamin D treatment as well as pc-TIPE1 transfection. *P<0.05, **P<0.01

Subsequently, we investigated whether the overexpression of TIPE1 could counteract the effects of vitamin D. Our results showed that compared to the glucose treatment group, vitamin D treatment led to a decrease in reactive oxygen species (ROS) production. However, the overexpression of TIPE1 reversed the effects of vitamin D under high glucose conditions by increasing ROS levels (Fig. 4A).

In this study, the analysis of autophagy flux demonstrated that the overexpression of TIPE1 resulted in a suppression of autophagy flux compared to the vitamin D group under high-glucose conditions (Fig. 4B). Additionally, the utilization of transmission electron microscopy revealed that the overexpression of TIPE1 led to a reduction in autophagosome formation compared to the vitamin D group under high-glucose conditions (Fig. 4C).

**Fig. 5:**
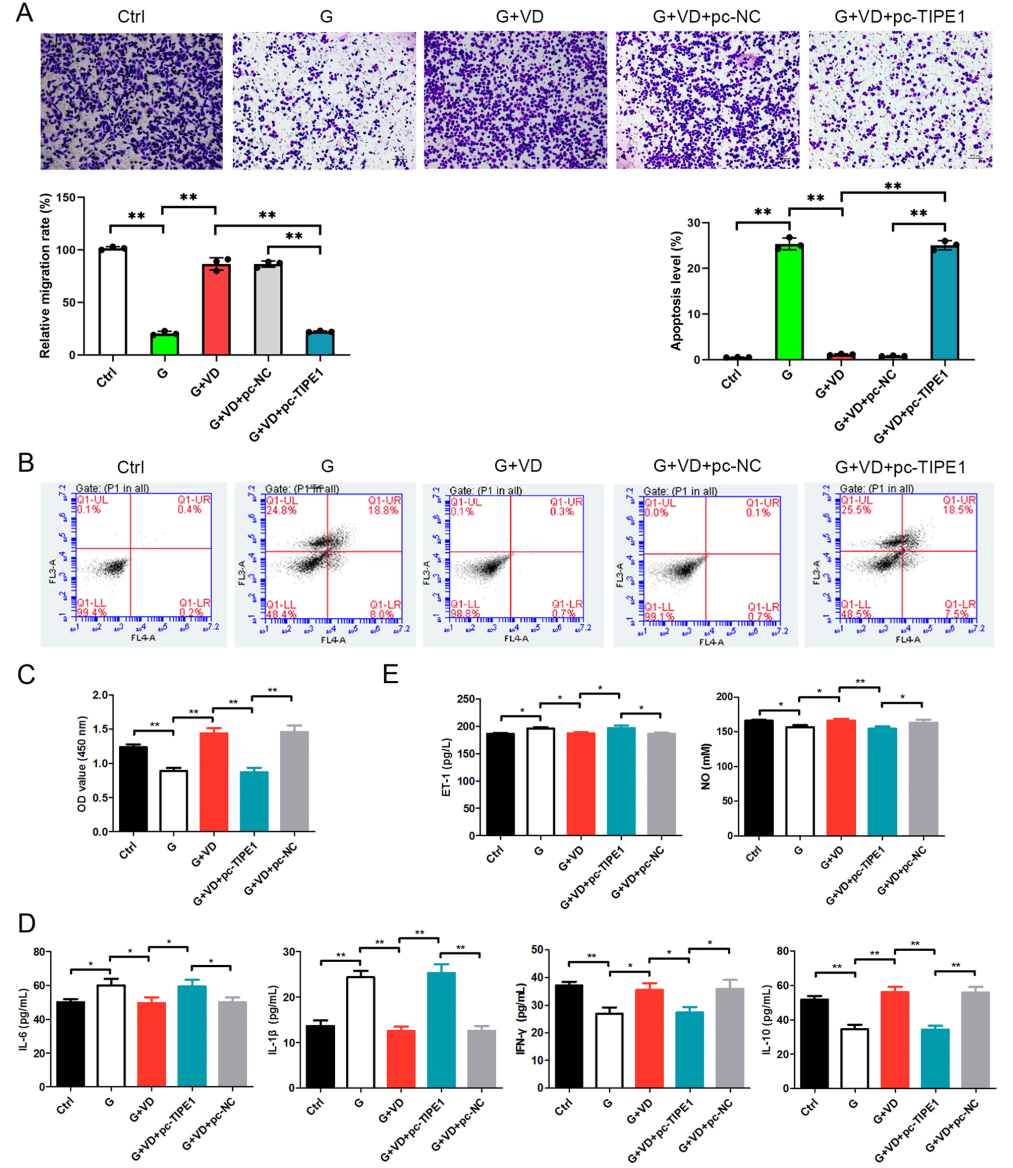
?Overexpression of TIPE1 reverses the effects of Vitamin D in apoptosis, migration and viability in human umbilical vein endothelial cells treated by high glucose. (**A**) Cell migration was tested by Transwell assay. (**B**) Cell apoptosis level was detected by flowcytometry. (**C**) Cell viability was analysed by CCK-8 assay. (**D**) ELISA was used to determine the level of inflammatory cytokines, (**E**) ELISA was used to test the level of ET-1 and NO. Ctrl, a normal concentration of glucose (5.6 mM); G, high glucose treatment; G+VD, high glucose plus vitamin D treatment; G+VD+pc-NC, high glucose plus vitamin D treatment as well as pc-NC transfection; G+VD+pc-TIPE1, high glucose plus vitamin D treatment as well as pc-TIPE1 transfection. *P<0.05, **P<0.01

Furthermore, the overexpression of TIPE1 exhibited a reversal of the effects induced by vitamin D under high glucose conditions, as evidenced by the inhibition of cell migration (Fig. 5A), the enhancement of cell apoptosis (Fig. 5B), the decrease in cell viability (Fig. 5C), and the promotion of inflammation (Fig. 5D). The upregulation of ET-1 and downregulation of NO were observed when TIPE1 was overexpressed in the presence of vitamin D supplementation (Fig. 5E). Additionally, treatment with vitamin D resulted in decreased expression levels of TIPE1 and reduced secretion of the inflammatory cytokine IL-6 in HUVECs exposed to high-glucose conditions.

### Serum vitamin D level was negatively associated with TIPE1 level in type 2 diabetes patients

**Fig. 6:**
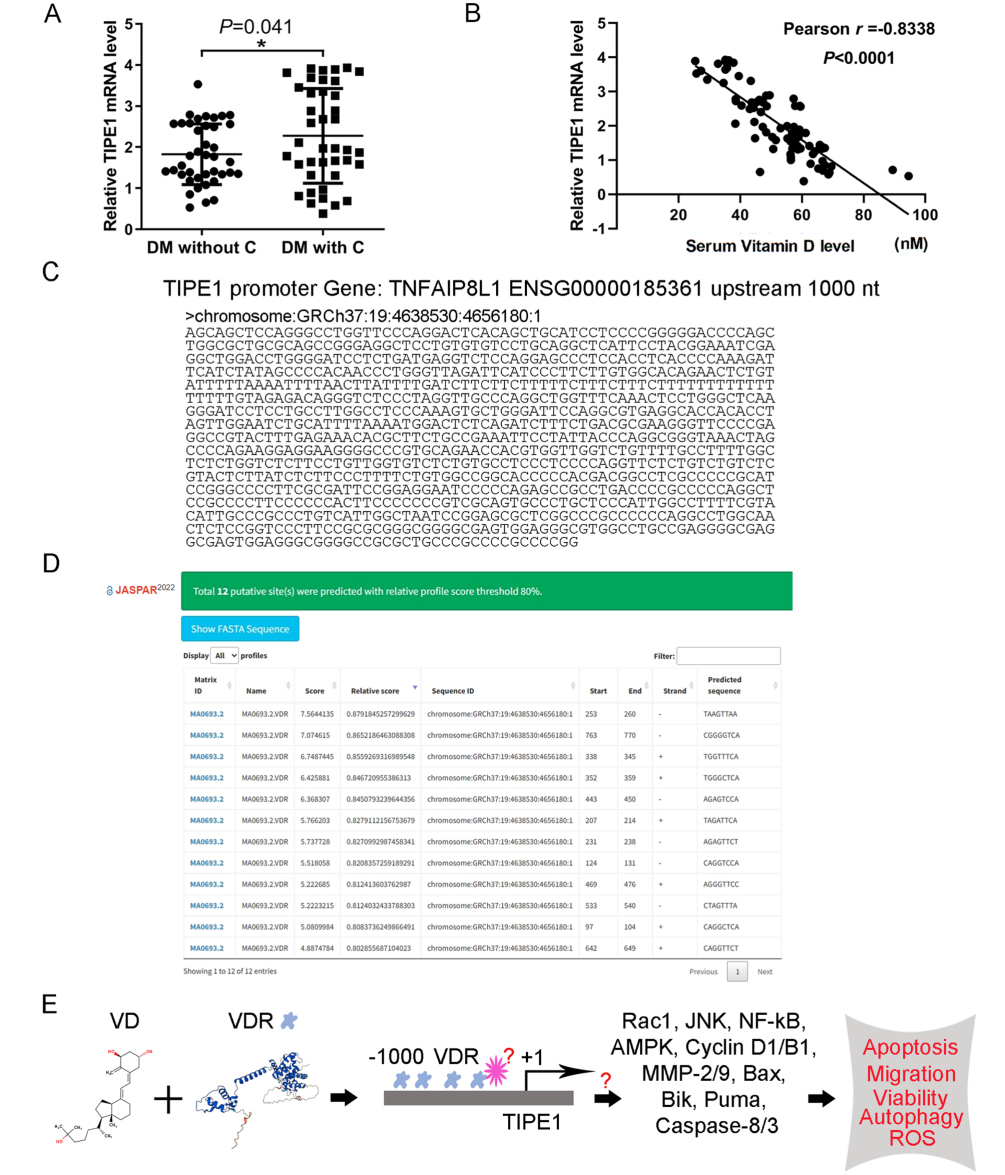
?Serum vitamin D level was negatively associated with TIPE1 level in type 2 diabetes patients. (**A**) Relative mRNA level of TIPE1 was detected in type 2 diabetes mellitus patients with or without microvascular complications by RT-qPCR. (**B**) Pearson correlation analysis of the mRNA expression of TIPE1 and serum vitamin D level in type 2 diabetes mellitus patients with or without microvascular complications. (**C**) The 1000 bp upstream promoter sequences of TIPE1 from the Ensemble website. (**D**) Potential binding sites of VDR on 1000 bp upstream promoter sequences of TIPE1 from the JASPAR website. (**E**) Possible function mechanisms of vitamin D on TIPE1 expressions. *P<0.05

In order to investigate the potential correlation between TIPE1 expression and vitamin D levels in patients, RT-qPCR was performed using serum samples from the same recruited patients depicted in Fig. 1. The findings of this study indicate that the relative TIPE1 mRNA expression was significantly elevated in diabetes mellitus patients with microvascular complications in comparison to those without complications, as depicted in Fig. 6A. Pearson correlation analysis revealed a negative correlation between TIPE1 mRNA level and serum vitamin D level in patients with diabetes mellitus (Fig. 6B).

We subsequently endeavor to elucidate the underlying mechanism by which vitamin D influences the expression of TIPE1. Typically, vitamin D engages with the Vitamin D receptor (VDR) to initiate VDR signaling. To accomplish this, we retrieve the 1000 bp upstream promoter sequences of TIPE1 from the ensemble website (Fig. 6C) and employ the JASPAR website to scan for potential binding sites of VDR on these promoters. The findings reveal the presence of 12 putative VDR binding sites on the TIPE1 promoter (Fig. 6D).

According to the report, TIPE1 exhibits a negative correlation with Rac1 and inhibits various pathways including JNK and NF-kB, cyclin D1/B1, and MMP−2/9. Additionally, TIPE1 upregulates Bax, Bik, Puma, and caspase−8/3, leading to apoptosis and reduced cell growth [17]. Furthermore, TIPE1 disrupts PHB2 mediated mitophagy [18]. Based on these findings, we propose a potential mechanism whereby vitamin D inhibits TIPE1 expression by binding with VDR and recruiting transcriptional repressors to the TIPE1 promoter. TIPE1 exerts its influence on various downstream signaling molecules, including Rac1, JNK, NF-kB, Cyclin D1/B1, MMP-2/9, Bax, Bik, Puma, and Caspase-8/3, thereby modulating the functions of HUVECs in the presence of elevated glucose levels (Fig. 6E).

## Discussion

In recent decades, research has demonstrated the impact of vitamin D deficiency on glucose metabolism, insulin secretion, and the development of type 2 diabetes [19]. Notably, a substantial proportion of individuals with type 2 diabetes exhibit a deficiency in vitamin D [20]. Our study has revealed a significant reduction in serum vitamin D levels among individuals with type 2 diabetes who experience microvascular complications, as compared to those without such complications. This finding suggests a potential association between vitamin D deficiency and the occurrence of microvascular complications in patients with type 2 diabetes.

Recent research indicates that the impact of elevated glucose levels on endothelial cells plays a significant role in the manifestation of clinical complications related to diabetic mellitus [21, 22]. The exposure of human umbilical vein endothelial cells (HUVECs) to high glucose levels results in an upregulation of the ET−1 gene expression and a decrease in cell viability [23]. Furthermore, the induction of apoptosis in vascular endothelial cells is heightened under high glucose conditions [24]. Notably, the administration of vitamin D treatment demonstrates promising outcomes in terms of enhancing cell viability, reducing mitochondrial reactive oxygen species (ROS) production, and suppressing mitophagy and inflammation in the presence of high glucose and particulate matter [25]. The study revealed that elevated glucose levels had a detrimental effect on the viability of HUVEC cells, leading to increased production of reactive oxygen species (ROS) and apoptosis, as well as a decrease in nitric oxide (NO) generation. Consequently, there was a reduction in the levels of antioxidant enzymes and an increase in proinflammatory cytokines. However, the detrimental impact of high glucose-induced endothelial oxidative injury was mitigated by the administration of 1,25(OH)2D3, which resulted in the upregulation of the Nrf2 antioxidant pathway [26]. In our investigation, vitamin D demonstrated a capacity to decrease cell apoptosis and enhance the migration and viability of endothelial cells exposed to high glucose.

The potential mechanisms underlying the effects of vitamin D on diabetes-related complications may involve its antioxidant, anti-inflammatory, and immune modulatory properties [27]. In the present study, we observed that vitamin D supplementation mitigated the damage to human umbilical vein endothelial cells (HUVECs) induced by type 2 diabetes mellitus, likely through the down-regulation of TIPE1. TIPE1 has been implicated in various cellular processes such as survival, migration, necroptosis, apoptosis, and autophagy [28–30]. Furthermore, it has been found to be upregulated in tubular epithelial cells of patients with diabetic nephropathy [18]. In vascular endothelial cells, the up-regulation of TIPE1 resulted in an increase in reactive oxygen species (ROS)-induced oxidative stress, leading to apoptotic cell death and the progression of atherosclerosis [10]. Additionally, the overexpression of TIPE1 reduced the levels of pAMPK and LC3B, thereby inhibiting autophagy in nasopharyngeal carcinoma cells [28]. Furthermore, the upregulation of TIPE1 in renal tubular epithelial cells in a high glucose environment disrupted mitophagy and facilitated the advancement of diabetic nephropathy [18]. In this study, we discovered that the overexpression of TIPE1 counteracted the effects of vitamin D on injury induced by high glucose in HUVECs.

Vitamin D supplementation has the potential to serve as a treatment for diabetes-related periodontitis by reducing oxidative stress and inflammation through the upregulation of Nrf2 signaling [31]. Ampelopsin has been found to protect endothelial cells from oxidative damage caused by hyperglycemia by inducing autophagy through the AMPK signaling pathway [32]. On the other hand, metformin has been shown to alleviate endothelial impairment resulting from hyperglycemia by downregulating autophagy through the Hedgehog pathway [33]. These findings indicate that both the induction and downregulation of autophagy have the ability to reverse injury caused by hyperglycemia. The data presented in our study suggest that vitamin D has the potential to mitigate the oxidative damage to endothelial cells induced by hyperglycemia through the induction of autophagy. In diabetic rats, the administration of vitamin D resulted in a reduction in ET−1 activity and an increase in NO levels [34]. Consistent with this, our investigation using HUVECs exposed to high glucose conditions demonstrated that vitamin D supplementation led to a decrease in ET−1 levels and an increase in NO levels. Additionally, a comprehensive analysis of intervention studies indicated that vitamin D supplementation significantly reduced fasting blood sugar, hemoglobin A1c (HbA1c), insulin concentrations, and homeostatic model assessment for insulin resistance (HOMA-IR) [35].

A subsequent meta-analysis demonstrated that the administration of vitamin D supplements resulted in improved glycemic measures and enhanced insulin sensitivity, thereby potentially serving as a preventive measure against the development of type 2 diabetes [27]. A comprehensive review and meta-analysis further confirmed that the supplementation of vitamin D led to an elevation in serum 25(OH)D levels and a reduction in insulin resistance. Notably, a substantial impact was observed when high doses of vitamin D were administered for a brief period, specifically targeting non-obese individuals of Middle Eastern descent who exhibited vitamin D deficiency or patients with optimal glycemic control at the outset [36]. Paricalcitol, a vitamin D analogue, was found to provide protection against hydrogen peroxide-induced injury in HUVEC by suppressing apoptosis [37]. In this study, we observed that the combination of vitamin D supplementation and insulin treatment resulted in improved diabetes biomarkers in type 2 patients with microvascular complications.

Collectively, our investigation revealed a diminished presence of serum vitamin D in individuals diagnosed with type 2 diabetes mellitus and exhibiting microvascular complications. Additionally, the administration of vitamin D supplements counteracted the detrimental impact of elevated glucose levels on human umbilical vein endothelial cells (HUVECs) by mitigating the expression of TIPE1 protein. Consequently, it can be inferred that vitamin D supplementation holds potential benefits for individuals with type 2 diabetes mellitus and microvascular complications.

## Data Availability

The datasets used or analyzed during the current study are available from the corresponding author on reasonable request.
